# Circulating Tumor Cells from Enumeration to Analysis: Current Challenges and Future Opportunities

**DOI:** 10.3390/cancers13112723

**Published:** 2021-05-31

**Authors:** Yu-Ping Yang, Teresa M. Giret, Richard J. Cote

**Affiliations:** 1 Department of Biochemistry and Molecular Biology, DJTMF Biomedical Nanotechnology Institute, Miller School of Medicine, University of Miami, Miami, FL 33136, USA; yyang22@med.miami.edu; 2 Department of Radiation Oncology, Miller School of Medicine, University of Miami, Miami, FL 33136, USA; tgiret@med.miami.edu; 3 Department of Pathology and Immunology, Washington University School of Medicine, St. Louis, MO 63110, USA

**Keywords:** circulating tumor cells, diagnosis, prognosis, therapeutic response, single-cell analysis, circulating tumor microemboli, ex vivo CTC culture

## Abstract

**Simple Summary:**

With estimated numbers of 1–10 per mL of blood, circulating tumor cells (CTCs) are extremely rare compared to white (a few million) or red (billions) blood cells. Given their critical role in metastasis, CTCs have enormous potential as a biomarker for cancer diagnosis, prognosis, and monitoring of treatment response. There are now efforts to characterize CTCs more precisely through molecular and functional analysis, expanding the CTC effort from one of diagnosis and prognosis to now include the use of CTCs to specifically target cancers and discover therapeutic solutions, establishing CTCs as critical in precision medicine. This article summarizes current knowledge about CTC isolation technologies and discusses the translational benefits of different types of downstream analysis approaches, including single-CTC analysis, ex vivo expansion of CTCs, and characterization of CTC-associated cells.

**Abstract:**

Circulating tumor cells (CTCs) have been recognized as a major contributor to distant metastasis. Their unique role as metastatic seeds renders them a potential marker in the circulation for early cancer diagnosis and prognosis as well as monitoring of therapeutic response. In the past decade, researchers mainly focused on the development of isolation techniques for improving the recovery rate and purity of CTCs. These developed techniques have significantly increased the detection sensitivity and enumeration accuracy of CTCs. Currently, significant efforts have been made toward comprehensive molecular characterization, ex vivo expansion of CTCs, and understanding the interactions between CTCs and their associated cells (e.g., immune cells and stromal cells) in the circulation. In this review, we briefly summarize existing CTC isolation technologies and specifically focus on advances in downstream analysis of CTCs and their potential applications in precision medicine. We also discuss the current challenges and future opportunities in their clinical utilization.

## 1. Introduction

Cancer metastasis, the process of tumor cells spreading from a primary tumor to distant organs, is the primary cause of cancer morbidity and mortality, which is responsible for about 90% of cancer-related deaths [1]. Circulating tumor cells (CTCs) detach from the primary tumor and invade surrounding tissue and travel to different sites through circulatory systems. These tumor cells shed into the bloodstream, ultimately settling and growing at distal organs throughout the body [2]. Because of their critical role as metastatic seeds, CTCs are becoming an essential landmark in cancer research [3,4]. However, it is difficult to identify CTCs from the blood of a patient with non-hematological cancers due to their extreme rarity, with numbers ranging from one to a hundred cells in a 7.5 mL tube of blood drawn [5,6]. Recent technical advancements in CTC enrichment have significantly improved the purity and recovery of CTCs from a patient’s blood, leading to the exciting prospect of a comprehensive investigation of CTCs [7]. Many studies have shown the potential of CTCs as a valuable prognostic and predictive biomarker in cancer management, helping to monitor the efficacy of therapies and detect early development of metastases via their downstream functional and molecular analysis [8,9]. Furthermore, CTCs are referred to as a liquid biopsy that offers a minimally invasive and real-time method as an alternative to tissue biopsy, an invasive procedure that presents only a “snapshot” of the tumor and is difficult to obtain over time [10,11].

In the following sections, we categorize existing CTC isolation technologies based on their distinct methods for isolating CTCs from other blood cells. We also describe the advances in downstream analysis of CTCs and their potential applications, such as single-cell analysis, CTC biobank, and CTC-associated cells in the blood. Finally, we address the current challenges and future opportunities in their clinical utilization.

## 2. CTC Isolation and Detection Techniques

In the past decade, we have witnessed remarkable improvements in the efficiency and accuracy of isolation of CTCs from peripheral blood samples. Several CTC isolation technologies are now commercially available and can overcome previous limitations, such as the rarity of CTCs in blood samples and the heterogeneity of CTCs. We summarize current techniques categorized by their use of CTCs’ physical properties (size, shape, density, and flexibility), biological properties based on tumor-specific markers expressed in the membrane of CTCs, and a combination of physical and biological properties using microfluidic devices (Table 1).

### 2.1. Isolation Based on Physical Properties

#### 2.1.1. Density-Based Separation

The differences in density of the diverse blood components have led researchers to use the density centrifugation method (Ficoll-Paque^®^) (CYTIVA, Marlborough, MA, USA) to separate peripheral blood mononuclear cells and CTCs from whole blood [12,13]. The OncoQuick^®^ system (Greiner Bio-One International GmbH, Kremsmunster, Austria) combines the density gradient centrifugation system and a porous barrier that increases the depletion of mononuclear cells [16]. The RosetteSep™ (STEMCELL Technologies, Vancouver, Canada) incorporates an immunoaffinity approach into the gradient centrifugation method by crosslinking unwanted PBMC cells to the red cells in the sample, forming immunorosettes that significantly improve the isolation efficiency for CTCs [17]. Recently, an integrated platform was developed (RareCyte Inc, Seattle, WA, USA) for enrichment, detection, and single-cell analysis of CTCs in a single workflow, through combining the AccuCyte CTC system for CTC collection based on density, together with CyteFinder (RareCyte Inc, Seattle, WA, USA), a scanning digital microscope with a six-channel fluorescence detection system, and CytePicker for individual CTC isolation [14,15]. These separation technologies are all based on the density of CTCs.

#### 2.1.2. Filtration-Based Separation

The first microfiltration system for CTC isolation was developed more than 50 years ago [48]. However, advances in track etching techniques have made filtration-based isolation methods more feasible [49]. The ISET^®^ filtration system (Rarecells Diagnostics, Paris, France) [20] and the ScreenCell^®^ system (ScreenCell, Sarcelles, France) are two commercially available systems for isolation and capture of CTCs onto a membrane for downstream analysis applications [21]. Recently, several automated filter-based isolation systems have been developed. The faCTChecker from Circulogix (Circulogix Inc, Hallandale Beach, FL, USA) is a microfilter technology composed of an automated fluid handler and a parylene-C membrane filter-based device for size-based capture and immunofluorescence analysis for identification of CTCs in the clinical setting [22,23]. Notably, unlike the track-etched polycarbonate filters, biocompatible parylene-C membrane filters are generated with microfabrication technologies and thus exhibit greater consistency of size, geometry, and density of the pores. These advantages give faCTChecker better capture efficiency and enrichment factor (seven-log depletion of leukocytes). Moreover, the faCTChecker is capable of live CTC capture by simply changing the geometry of the pores on membrane filters, which reduces flow resistance to prevent damage of captured viable cells [50]. Releasing captured cells from the faCTChecker is also achieved by coating temperature-responsive polymers on filters, which enables the detachment of cells as the temperature increases [51]. The Parsortix^®^ technology (ANGLE, Surrey, UK), a microfluidic-based platform can capture and harvest CTCs from the blood using a filtration cassette, which is based on cell size and deformability [24,25]. Staining of the captured cells can be performed directly in the filtration cassette. Because the filtration cassette replicates the dimensions of a microscope slide, CTCs can be visualized under a microscope and analyzed by cytological, histological, or immunofluorescent staining techniques [52]. Additionally, the system enables the capture of viable CTCs and later release of the captured cells from the filtration cassette for further downstream analysis, such as single-cell analysis and ex vivo CTC culture.

#### 2.1.3. Contactless Separation

Since filtration-based methods may cause mechanical damage to CTCs, thus affecting further downstream analysis, several contactless separation methods have been developed to avoid the damage to CTC viabilities during the enrichment process. The VTX-1 Liquid Biopsy System (Vortex Biosciences, Pleasanton, CA, USA) uses a microfluidic approach that does not use any antibodies or microfilters to capture cells; instead, it uses two types of forces: a shear-gradient lift force and a wall-effect lift force that directs particles away from the wall of the microfluidic channels where the blood is injected. The cells are passively separated by size through the application of inertial forces in the microfluidic device that affect the cells’ positioning within the flow channel. In addition, inertial microfluidics generate microvortices that trap the larger tumor cells while allowing the smaller blood cells to pass. The tumor cells are trapped in the microfluidic device until released from the chip by lowering the flow rate [26]. Another inertial focusing platform is the ClearCell^®^ FX1 automated system (Biolidics Limited, Mapex, Singapore), a label-free CTC enrichment that uses a microfluidic principle with the inertial migration of particles and a secondary flow in curvilinear channels, which allows the CTChip^®^ FR (Biolidics Limited, Mapex, Singapore) to separate the larger cancer cells from the blood cells [27]. The dielectrophoresis (DEP) technique, the movement of particles or cells under the effect of an uneven electric field, has also been used for continuous separation of CTCs. The separation is based on the heterogeneity of the cells in structure and conductivity and on an electric field exerting a positive force (pDEP) and negative force (nDEP) on the different cells within a dielectric affinity column [53]. The separation is performed via migration mechanism, using pDEP to attract CTCs and nDEP to repel leukocytes; the ApoStream^®^ technology (Precision for Medicine, Bethesda, MD, USA) leverages differences in the dielectric properties of the cell, capturing cancer cells with positive DEP [28]. Another commercial system, DEPArray™ (Menarini Silicon Biosystems, Bologna, Italy) separates single cells by retention mechanism and the use of nDEP forces to trap CTCs in deep cages by the gentle dye electrophoresis force [54]. Another example of the electrophoresis-based separation method that uses electrophoresis and dielectrophoresis (DEP) forces within an electrical field is a new microfluidic device. The microfluidic system is capable of separating circulating tumor cells from normal peripheral blood based on their physical properties, particularly morphological and biophysical differences, such as membrane capacitance, shape, size, and conductivity [55].

### 2.2. Isolation Based on Biological Properties

The isolation and capture of CTCs based on biological characteristics of those heterogeneous cells utilize different biological markers expressed in the cells (Table 2) [18,19,29,30,31,32,33,34,35,36,37,38,39,40,41,42,43,45,56,57,58,59,60,61,62]. The first data describing the detection and characterization of carcinoma cells in the systemic circulation (bone marrow) based on cytokeratin and MUC-1 glycoprotein expression in epithelial cancer cells, but not in healthy cells, were published 25 years ago [63]. This observation was the basis for the development of the CellSearch^®^ system technology (Menari Silicon Biosystems, Bologna, Italy), the first FDA-cleared technology for CTC detection for metastatic breast, prostate, and colon–rectal cancer. The system is based on the positive enrichment of CTCs by immunoaffinity, using nanoparticles with antibodies targeting the epithelial cell adhesion molecule (EpCAM) antigens for CTC capture. After enrichment, fluorescent reagents are added for identification and enumeration of CTCs of epithelial origin that exhibit the phenotypes EpCAM positive; cytokeratins 8, 18, and 19 positive; and CD45 negative, a marker specific to leukocytes. A DNA stain, DAPI (4′,6-diamidino-2-phenylindole) fluorescence, is also added to identify the nuclei of both CTCs and leukocytes. The limitation of this technique is its dependence on the expression of EpCAM [44].

Another positive enrichment method, AdnaTest (Adnagen GmbH, Langenhagen, Germany), uses a combination of three different antibodies that bind to different antigens. This method detects gene transcripts of tumor markers EpCAM, the transmembrane mucin 1 (MUC-1) protein, and the human epidermal growth factor receptor 2 (HER2) using a multiplex reverse transcription polymerase chain reaction (RT-PCR) [64]. The method is interpreted as CTC positive if at least one or more of the three markers show an expression of signal intensity equal to or greater than 0.15 ng/μL, a threshold suggested by AdnaGen.

The magnetic-activated cell sorting system (MACS) is a method of separation of different cell populations depending on their surface antigens or cluster of differentiation (CD) molecules [65]. The CTCs are captured and isolated by immunolabeling with the superparamagnetic particles conjugated with antibodies for the enrichment and further characterization by immunocytologic, molecular, and cytogenetic assays [46,66]. A positive enrichment that uses the immunomagnetic approach is the cell separator MagSweeper (Illumina Inc, San Diego, CA, USA), a robotic liquid biopsy device that isolates and purifies live CTCs using the EpCAM protein as a biomarker, excluding cells that are not bound to magnetic particles [67]. The workflow assures high purity, efficiency, and viability for subsequent gene expression studies.

### 2.3. Isolation Based on a Combination of Physical and Biological Properties

Microfluidic-based enrichment technologies have been explored for CTC detection methods. The first microfluidic device or affinity-based microchip system is the CTC-chip, which consisted of an arrangement of microposts where the CTCs are captured as the whole blood is pushed over the surface of the chip. The platform captures and analyzes the target cells by using antibody-based separation, with antibodies such as anti-EpCAM [47]. The second-generation platform called CTC-iChip is also a system composed of two modules: the first uses continuous deterministic lateral displacement for the size-based separation of RBCs, platelets, and other blood components from the white blood cells (WBC) and CTCs; the second uses inertial focusing to align the larger cells and facilitate the subsequent separation by magnetophoresis, acquiring the cell of interest and depleting the white blood cells and contaminants [68].

The geometrically enhanced differential immunocapture (GEDI) chip is a technique to isolate CTCs using size-dependent collision frequency that maximizes the CTC–wall interactions while minimizing the interactions of other blood cells and antibody-coated 3D posts. This technique can achieve high efficiency and high-purity capture as it allows for the combination of more than one antibody marker [69]. Another system, OncoCEE^®^ (Biocept Inc, San Diego, CA, USA), uses the technology of random distribution of posts to disrupt the laminar flow. It uses a Biocept^®^ antibody capture cocktail (Biocept Inc, San Diego, CA. USA) against tumor-associated antigens from cancer cells of both epithelial and mesenchymal phenotype, as well as cancer stem cells [70]. The IsoFlux System™ consists of microfluidic cartridges. This system is based on immunomagnetic separation using anti-EpCAM magnetic beads after the selection of cells through the leukapheresis technique from the buffy coat sample [71]. The LiquidBiopsy^®^ platform (Lunglife AI Inc, Oaks, CA, USA) is a high throughput CTC flow cell-associated automated system that continuously flows liquid through a laminar flow cell using a programmable logic-controlled pump and a pipetting arm. The system is composed of a three-layer sheath flow for a positive selection of the CTC population from whole blood using antibody beads anti-EpCAM, anti-MelCAM (melanoma cell adhesion molecule), anti-HER2, anti-MUC-1, and anti-TROP2 or TACSTD2 (tumor-associated calcium signal transducer 2) [72].

The affinity-based microchip system, which consists of an arrangement of microposts, is a more complex microfluidic device system when compared to the surface-based microfluidic technology. An example of this technology is the microvortex-generating herringbone-chip (HB-chip), which applies a passive mixing of blood cells through the generation of microvortices to increase the interaction between the CTCs and the antibody-coated chip surface [73]. Sheng and collaborators developed a new form of the HB-chip, the geometrically enhanced mixing (GEM) chip, that improved the previous system by increasing the groove width for high efficiency and high-purity tumor cell capture [74]. Another surface-based affinity system is the graphene oxide (GO) chip. It is assembled using functionalized graphene oxide nanosheets on a patterned gold surface that allows CTC capture with high sensitivity at a low concentration of target cells [75]. The microfluidic platform, presented by BioFluidica (BioFluidica Corp., San Diego, CA, USA), is a modular CTC sinusoidal microsystem composed of three modules: a thermoplastic CTC selection module comprised of sinusoidally-shaped channels that contain anti EpCAM antibodies; an impedance sensor module for label-free CTC counting; and a staining and imaging module for identification and characterization of the CTCs [76].

The NanoVelcro CTC chip (CytoLumina Technologies Corp., Los Angeles, CA, USA) is composed of an overlaid polydimethylsiloxane (PDMS)-based chaotic mixer, anti-EpCAM antibody-coated silicon nanowires (SiNW), and a multilayer chip holder that assemble the functional components to immobilize CTCs. The system engenders vertical flows of the blood sample with a considerable speed that enhances the contacts between CTCs and the capture substrate, preserving cell morphology [77,78]. Another immunoaffinity-based form includes the negative enrichment technology that uses antibodies against the common leukocyte antigen CD45, a pan-leukocyte surface marker. Many other groups have reported a CK-positive/CD45-positive or double-positive cell phenotype in metastatic cancer patients [73,75,79]. Some of the commercially available options are The EasySep™ Human CD45 Depletion Kit (STEMCELL Technologies, Vancouver, Canada) [80] and the quadrupole magnetic separator (QMS), composed of an immunomagnetic sorting system that uses an automated cell counter, filtration, and visual counting or a cytospin for cell analysis [81].

Expanding the variety of detection methods and the including markers that help to characterize cell populations at different phenotypic stages is crucial for improving cancer cell detection. The low abundance in blood and limited blood volume accessible from a cancer patient may represent a significant constraint on the detection of a particular cell population, such as the CTCs and their clusters. To address those limitations, nanotechnology approaches have been proposed to identify and isolate these cells. For example, with the ability to report a protein expression profile for CTCs as a function of surface marker expression, magnetic ranking cytometry (MagRC) has achieved a high level of sensitivity and resolution. The microfluidic chip used for MagRC contains 100 distinct zones with varied magnetic capture zones. An array of X-shaped structures generates regions of low velocity, and circular nickel micromagnets within the channel enhance a magnetic field that is applied externally, promoting efficient CTC capture [82]. In addition, a new strategy for detection of CTC and single-CTC proteomics was described in estrogen receptor-positive (ER+) breast cancer patients using the microfluidic single-cell resolution Western blot method for a panel of protein expression [83].

Recent advances in nanomaterials and nanotechnology offer high sensitivity, specificity, and multiplexed measurement capacity in CTC isolation and detection [7,84,85]. For example, the gold nanoparticles (AuNPs) have a strong binding capacity and the ability to be easily synthesized, prompting their use as an option for detection and capture of CTCs. They will serve as a good platform for assembling aptamers for high efficiency cell capture [86]. Other 3D nanosurfaces used as ultrasensitive platforms in CTC detection are silicon nanowires [87], graphene oxide [75], carbon nanotubes [88], and polymer nanofibers [89].

### 2.4. In Vivo CTC Detection

In addition to in vitro isolation of CTCs, several techniques and devices have been developed and show the potential for in vivo applications (e.g., in vivo CTC collection and direct detection). GILUPI CellCollector^®^ (GILUPI GmbH, Potsdam, Germany) contains a Seldinger guidewire functionalized with EpCAM for capturing live CTCs when applied in the peripheral vein. This technique allows the flow of up to 1.5 L of blood, and the collected CTCs are characterized by immunocytochemical staining [90]. Recently, Kim et al. [91] developed a wearable in vivo indwelling intravascular aphaeretic CTC isolation device to continuously collect CTCs directly from a peripheral vein, showing its capability to screen 1–2% of the entire blood over 2 h in canine models. However, these aforementioned devices still need to be placed into the peripheral vein, which is considered invasive, although minimally. In this regard, direct detection of CTCs in patients without an invasive procedure is an ideal approach in clinical settings. Nolan et al. demonstrated that GFP^+^ CTCs can be directly detected by using in vivo flow cytometry (IVFC) in animal models [92]. Moreover, the recent development of Cytophone technology (University of Arkansas for Medical Science, AR, USA) shows direct detection of CTCs in patients with melanoma, based on the detection of melanin-bearing CTCs by using an in vivo photoacoustic flow cytometry [93].

## 3. Downstream Analysis of Circulating Tumor Cells

CTC counts have shown to have a promising prognostic value in monitoring cancer progression and therapeutic response for advanced metastatic patients. The efficacy of using CTC counts as a robust independent biomarker in clinical settings, however, is still under investigation. The most important limitation is that there is no gold-standard CTC approach that is widely agreed upon in the CTC community. Since CTC isolations and enumerations are conducted in different laboratories using different techniques, they result in disparities in CTC quantification depending on the method of choice. Moreover, CTCs are extremely rare in early-stage cancer patients and in advanced metastatic patients. For these patients, employing CTC counts alone as a prognostic marker may introduce biases in the early prediction of the outcome. Other factors can also affect the baseline CTC count of cancer patients. Inflammatory breast cancer patients and small-cell lung cancer (SCLCL) patients with chronic obstructive pulmonary disease (COPD), for example, may have a greater chance of releasing tumor cells into the bloodstream than other types of cancer patients due to high vascularity and increased microvessel density [94,95].

**Figure 1:**
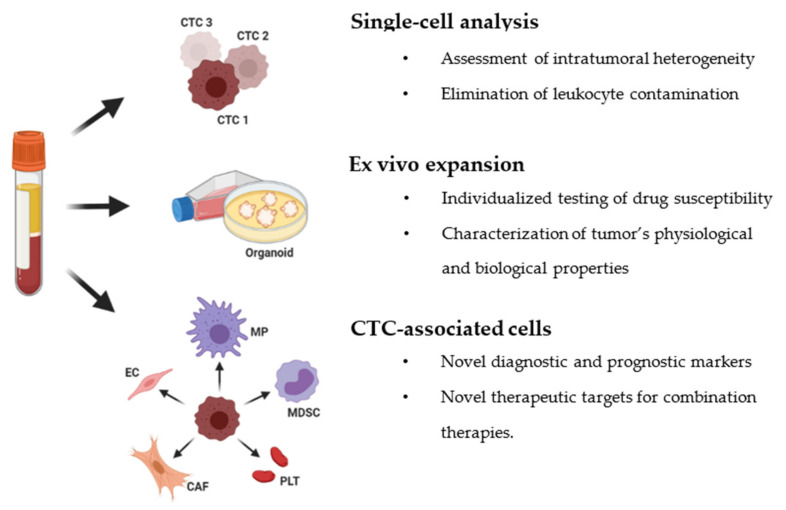
Current approaches for downstream molecular and functional analysis of circulating tumor cells. (CTC: Circulating tumor cells; MP: Macrophage; MDSC: Myeloid-derived suppressor cell; EC: Endothelial cell; CAF: Cancer-associated fibroblast; PLT: Platelet).

Currently, CTC research is extending beyond enumeration for more accurate cancer diagnostics. Immense efforts have been devoted to a genotypic and phenotypic analysis of CTCs, such as molecular characterization, ex vivo expansion, and investigation of crosstalk between CTCs and their associated cells (e.g., stromal cells and immune cells). The information obtained from these studies provides unprecedented insights into the metastatic process and allows us to explore prognostic biomarkers and therapeutic targets for cancer management (Figure 1).

### 3.1. Single-Cell Molecular Analysis of CTCs

Identifying molecular defects associated with cancer progression and drug resistance is essential for the improvement of cancer diagnostics and therapeutics. Although molecular interrogation of CTCs holds great promise as a noninvasive “liquid biopsy” for real-time monitoring of the change of cancer drivers, it may also introduce a strong bias in the molecular analysis due to the extremely rare events of CTCs in the impure samples, even after an enrichment process. As a result of recent advances in technology (e.g., next-generation sequencing and single-cell isolation), researchers are now able to perform in-depth molecular characterization of CTCs at the single-cell level, which provides more clinically useful information when studying CTC heterogeneity and improving early patient stratification [96]. Of the biological materials used for “omic” analysis, genomic DNA is the most stable and unchangeable biomaterial during a stringent CTC enrichment process. Given the very limited DNA quantities of a single cell, whole genome amplification (WGA) is required for comprehensive genotyping analysis, such as comparative genomic hybridization (CGH) and next-generation sequencing (NGS) [97,98]. These techniques have been used to identify and monitor potentially actionable mutations that drive cancer development and resistance to therapy. For example, in small-cell lung cancer, copy number variations (CNVs) were identified in CTCs to determine chemosensitive versus chemorefractory patients [99]. Additionally, the comparison of the copy number alternations (CNA) between single primary tumor cells and circulating tumor cells reveals the focal CNAs affecting the MYC gene and the PTEN gene, which are the drivers for cancer metastasis [100].

In comparison with genomic analysis, transcriptome analysis provides more detailed information that allows us to precisely identify cancer drivers for diagnosis and therapeutic targets [101]. Although CTC enrichment steps may cause an effect on gene expression, researchers are still able to identify key genes by analyzing their RNA contents from different types of cancer patients, determining which expression is associated with cancer progression and treatment response [102,103,104,105,106,107]. For example, a non-canonical Wnt pathway has been identified in a subpopulation of prostate CTCs that involve resistance to androgen receptor (AR) target therapies. Similar work has been performed in pancreatic cancers, showing that expression of Wnt2 regulates a metastasis-associated survival signal pathway that increases metastatic propensity. Importantly, various recent microfluidic-based platforms have been developed, which enhance the efficiency in single-cell sorting [108,109,110,111,112]. Several commercial devices enable automatic isolation and analysis of individual CTCs in a single workflow, such as the Polaris system (Fluidigm), Chromium (10X Genomics), and DEPArray system (Siliconbiosystems) [113,114].

In addition to the investigation of DNA and RNA biomaterials, researchers are also investigating the important roles that microRNAs and epigenetic modification play in mediating gene expression (e.g., oncogenes and tumor suppressor genes). Thus, profiling microRNA and DNA methylation in CTCs can also make a valuable contribution to cancer prognostics [115,116]. Unlike single-cell genomics and transcriptomics, high-throughput proteomic analysis at the single-cell level is challenging due to the requirement of large amounts of proteins for analysis. While the limitation impacts single-cell proteomics, mass cytometry has enabled the measurement of up to 40 different target proteins in single CTCs. Mass cytometry uses antibodies conjugated with heavy metal isotopes to label cellular proteins, which allows for precise quantification of target proteins at the single-cell level [117]. Moreover, another technique has been developed for profiling protein expression in single CTCs using microfluidic Western blotting [83].

### 3.2. Ex Vivo Expansion of CTCs

Researchers have widely used established cancer cell lines for studies of cancer pathological mechanisms and anticancer drug testing. Despite their significant role in cancer research, cancer cell lines do not completely mimic the original solid tumors [118]. Instead, the primary culture of patient-derived cancer cells can be a good model for identifying cancer markers and predicting the drug response of individual patients’ tumors. Based on the mechanism of the metastatic cascade, CTCs offer valuable information about metastatic development over primary tumor cells obtained from a piece of tumor tissue. Therefore, ex vivo expansion of CTCs is another potential model to aid treatment decisions in the metastatic setting.

Although the establishment of cell lines from CTCs has been challenging due to the rarity of CTCs in blood, significant advances in viable CTC enrichment techniques and culture systems have been making it possible. In 2005, Alix-Panabieres et al. developed the EPISPOT assay method, which enables a short-term culture of CTCs ex vivo from cancer patients [119]. In 2013, the first permanent CTC cell lines were established from patients with advanced-stage breast cancer [120]. Interestingly, only 3 out of 38 patients’ CTCs were able to generate CTC cell lines, and these CTC cell lines share the same genetic defects (HER2+, EGFR+, HPSE+, and Notch1+) and pathological phenotype (brain metastasis). Later, Yu et al. isolated CTCs from ER+ breast cancer patients using a CTC-iChip microfluidic device and propagated these isolated CTCs in culture [121]. These generated CTC cell lines (6 out of 36 patients) were maintained for more than six months under hypoxic conditions with a combination of cocktail of growth factors and low attachment plates. They also performed drug sensitivity testing for these CTC cell lines, which revealed potential new therapeutic targets. CTCs can also be co-cultured with stromal cells for maintaining their life span in vitro. Zhang et al., captured CTCs from patients with lung cancer using a microfluidic device, and these captured CTCs were cultured in situ along with tumor-associated fibroblasts and extracellular matrix [122], showing a model of the tumor microenvironment and subsequent ex vivo expansion of CTCs.

Cancer drug screening in patient-derived tumor cells holds great promise for translational and personalized medicine. Developing a physiologically relevant cell culture system is critical to the advancement of accurate and efficient identification of drugs. Recently, researchers have developed a novel culture system, known as organoids, which can mimic many structural features and pathophysiological functions of tumors [123,124,125]. In organoid culture systems, single cancer cells are automatically grown and differentiated into original tissue architecture embedded in a 3D extracellular matrix (laminin and collagen) along with stem cell niche factors. Organoids have been successfully generated from primary tumors, metastatic lesions, and CTCs, which can be used in high-throughput screening assays for biomarker and drug testing [126,127]. Gao et al. established organoid cultures from metastatic biopsies and CTCs from patients with metastatic prostate cancer [128]. These patient-derived organoid lines harbor genetic defects similar to parental prostate cancer, which can be used as genetically manipulatable models for drug response.

### 3.3. Role of CTC-Associated Cells

Circulating tumor microemboli (CTM), so-called CTC clusters, the heterogeneous multicellular clumps that are released from the primary tumor, have been shown to have higher metastatic potential than single CTCs in animal and clinical studies [129,130]. CTMs exhibit high resistance to apoptosis induced by anoikis, shear stress, and chemotherapy. They also can escape host immunosurveillance and are easy to trap in a small blood vessel capillary [131]. Other than tumor cells, the overall CTM is composed of different types of cells, such as stromal cells and immune cells. Recently, it has been reported that plakoglobin is a vital cell adhesion molecule that mediates the integrity of CTMs in breast cancer. Interestingly, expression of plakoglobin specifically occurred at the certain regions of primary tumors where the blood vessels are surrounded. This study suggests that CTMs originate from the detachment of primary tumors [129]. In addition to tumor cells, these non-tumor components have demonstrated their contribution to the survival and metastatic advantages of CTMs. Thrombocytosis is a significant unfavorable prognostic factor in many types of cancer and is highly observed in cancer patients with metastasis [132]. Growing evidence has shown that CTCs may use platelets as a protective shield to escape from host immune surveillance and as facilitators to enhance the attachment to endothelial cells at the metastatic site [133,134,135]. Recently, researchers have been able to isolate platelet-covered CTCs using a herringbone micromixing device (HB-chip) functionalized with a CD41 antibody against human platelets [136]. Notably, the CD41 HB-chip showed high capture efficiency of CTCs in comparison with the EpCAM HB-chip, suggesting platelet-covered CTCs are the dominant CTC subpopulation in blood. Based on clinical observation, strategies to eradicate CTCs using platelets as therapeutic tools have been developed [137,138]. For example, Li et al. [137] coated nanoparticles with platelet membrane-derived vesicles and showed that these platelet membrane-functionalized particles can target CTCs and reduce metastasis in animal models. The same group also developed genetic engineering platelets to neutralize circulating tumor cells [138].

The tumor microenvironment is a complex ecological system where tumor cells, stromal cells, infiltrating immune cells, and extracellular matrix, as well as the blood and lymphatic vascular networks, together orchestrate cancer metastasis. In the primary tumor, tumor-associated macrophages (TAMs) have been well studied as critical metastatic effectors and demonstrated to stimulate angiogenesis and promote tumor cell proliferation, invasion, and intravasation [139]. In blood, circulating macrophages have been identified and shown to associate with CTCs [140,141]. Interestingly, those CTC-associated macrophages show a large overall size (21–300 µm in length), and the number of those cells in the blood is increased in patients treated with chemotherapy [140]. Interestingly, recent discoveries show that CTCs can fuse with macrophages, and these fusion cells play an important role in tumor heterogeneity and chemoresistance [142]. In addition to circulating macrophages, myeloid-derived suppressor cells (MDSCs) have been shown to facilitate cancer metastasis by shielding CTCs from immune surveillance [143]. Clinical evidence has shown higher numbers of circulating MDSCs in cancer patients compared to healthy donors, suggesting that MDSCs have potential as a prognostic marker [144,145,146].

Tumor-associated stromal cells can also significantly contribute to the tumor microenvironment, acting as feeder cells to support tumor growth and development [147]. These feeder cells secrete various growth factors, chemokines, and cytokines that can promote proteolytic remodeling of extracellular matrix, collective migration, tumor vasculatures, invasion, drug resistance, and evasion of immune surveillance. During intravasation, cancer-associated fibroblasts (CAFs) have been shown to degrade extracellular matrix, creating tunnels that allow tumor cells to pass through and lead to blood vessels. Growing evidence shows that CAFs are clustered with CTCs in blood, and a high number of CAFs are found in patients with metastatic disease [129,148]. Circulating endothelial cells are other critical stromal cells that associate with CTCs in the blood and protect them from anoikis-induced apoptosis. Yadav et al. showed that Bcl-2-overexpressed circulating endothelial cells were released from primary tumors and co-migrated with CTCs to distal sites in an animal model. The interaction between the two types of cells enhanced anoikis resistance via the activation of the Src/FAK pathway [149].

## 4. Current Challenges and Future Direction for Clinical Utility

Innovative approaches have dramatically expanded the CTC field in recent years. Although promising results have revealed the potential clinical value of CTCs, the utility of CTC tests in clinical settings still faces significant challenges. To achieve the same widespread clinical utility as other diagnostic tests, such as pregnancy or blood glucose tests, several limitations need to be overcome. Based on the current standard procedure of CTC tests, one of the challenges comes from CTC sampling where 7.5 mL of blood sample is withdrawn from venous blood vessels. Because CTCs are extremely rare in the blood, it is critical to consider whether the sample size (7.5 mL) accurately reflects the total of 5 L of blood in an adult human. The evidence has shown that increasing the sample volume from 7.5 mL to 30 mL significantly improves the CTC detection rate from 13% to 47%. This indicates that a large sample size or multiple blood drawing may be the essential requirement for CTC tests in clinical settings in the future [150]. Another concern of CTC sampling is whether a greater number of CTCs can be detected in arterial blood or venous blood. Terai et al. [151] provided evidence that CTCs were detected in all patients when arterial blood was analyzed, whereas only half of the patients showed positive for CTCs by using venous blood. These findings suggest that arterial blood may be a better source of CTCs than venous blood. Furthermore, collecting and shipping blood samples without compromising CTC yield and viability is also critical for the detection and downstream analysis of CTCs. Currently, blood samples are collected in CellSave tubes containing a fixative solution that, although it enables storage of blood samples, causes cell death and crosslinking of intracellular contents of CTCs that affect functional analysis, especially single-cell CTC characterization or ex vivo expansion of CTCs. Recently, Wong et al. developed an approach for whole blood stabilization, preserving whole blood in an unfixed, viable state for up to 72 h [152].

Technological advancements have led to the development of many innovative CTC assays that can significantly improve CTC detection; however, this variety has led to discordance in the CTC community due to methodological discrepancies. Therefore, the requirement of clinical validity in larger studies for these new CTC detection techniques is essential for future clinical utility. Other technical factors may also contribute to the limitations of CTC tests in clinical settings. For example, process time and procedure may cause damage to CTCs, resulting in variability and bias for data interpretation. Furthermore, accurate identification of CTCs remains an enormous challenge owing to tumor heterogeneity. Concerning the CellSearch^®^ system, using anti-EpCAM antibody-conjugated magnetic beads isolates CTCs followed by confirming CTCs using pan-cytokeratin (Pan-CKs). However, downregulation of epithelial markers on CTCs, such as EpCAM, is associated with epithelial–mesenchymal transition (EMT), a biologic progress where epithelial cells acquire a mesenchymal phenotype and thus become more aggressive and invasive. Thus, the CellSearch^®^ system is unable to isolate EpCAM negative CTCs. As a result, the system underestimates the number of CTCs and only detects them in about 60% of metastatic breast cancer patients. Moreover, CK-negative CTCs can often be found in patients associated with poor prognosis. Therefore, there is an unmet need for the investigation of specific CTC biomarkers. The researchers have used cell surface vimentin, a well-known EMT marker, to isolate CTCs and showed that EMT CTCs correlate with disease progression or relapse of cancer patients [153,154]. Notably, given that EMT-related biomarkers are also expressed on the surface of certain types of normal cells (e.g., fibroblasts, monocytes, and macrophages), it is imperative to identify specific and broad markers for isolation and characterization of CTCs. For example, a recent study identified oncofetal chondroitin sulfate (ofCS), which is only expressed by placental cells and both epithelial-type and mesenchymal-type cancer cells, but not in blood cells, and tightly binds to the malaria VAR2CSA protein (rVAR2). This finding made rVAR2 an interesting and efficient tool for the isolation of CTCs especially [155]. A multi-marker strategy is another approach for isolating and identifying CTCs with heterogeneous phenotypes [156,157,158,159]. For example, combining EpCAM, HER2, EGFR, and MUC-1 enables the identification of 99.2% of cancer patients [158]. Interestingly, a novel circulating hybrid cell (CHC) population has been identified in patients, which possesses both hematopoietic and epithelial tumor properties [160]. This finding might prompt scientists to review the classical definition and isolation of CTCs in human cancer, excluding any CD45-expressing cells. Regarding the clinical practice of CTC tests in the future, more efficient enrichment techniques and larger panels of detection markers will be explored to avoid losing assay specificity and sensitivity, as well as damaging intracellular contents of CTCs.

## 5. Conclusions

Early detection of cancer is critical for the reduction of cancer morbidity and mortality. CTCs have recently emerged as a surrogate marker, and screening for CTC has the potential not only for early cancer diagnosis but also for real-time monitoring of disease progression and therapeutic response. In addition to CTC enumeration, downstream analysis of CTCs provides valuable insight into mechanisms of cancer metastasis, which allows us to identify targets for the most effective pharmaceutical treatments. In the future, although utilization of CTCs in clinical practice still has many limitations that require a high level of clinical validity, a combination of CTCs with other liquid biopsy partners, such as cell-free DNA and exosomes as an alternative to traditional biopsies, will pave the road for early cancer diagnosis and personalized treatment.

## Figures and Tables

**Table 1:** CTC enrichment technologies.

Technology	Detection Method	Description	References
The Ficoll-Paque^®^	Centrifugation	Cell separation based on relative density	[12,13]
AccuCyte CTC		Special device for collecting buffy coat that further spreads on a slide for staining CTCs	[14,15]
OncoQuick^®^	Centrifugation/filtration	Enriched CTCs in the interphase between the porous barrier and the separation medium	[16]
The RosetteSep™ CTC Enrichment Cocktail	Immunoaffinity centrifugation	Standard density gradient centrifugation combined with tetrameric antibody complexes for removing blood cells (negative selection)	[17,18]
Quadrupole Magnetic Separator (QMS)	Immunoaffinity/filtration	The flow channels and the permanent magnet assembly for negative selection of CTC enrichment	[19]
The ISET^®^	Filtration	Isolation of CTCs and CTM based on size	[20]
ScreenCell^®^ System		Size exclusion technology for isolating circulating rare cells (CRCs)	[21]
faCTChecker—Circulogix		Automatic filtration-based CTC enrichment system	[22,23]
Parsortix™	Microfiltration	Microfluidic technology that uses a cassette and captures CTCs based on their less deformable nature and larger size	[24,25]
The VTX-1 Liquid Biopsy System		Laminar microvortices to isolate and concentrate CTCs	[26]
ClearCell^®^ FX1 System and CTChip^®^ FR		Under the influence of inherent centrifugal forces, the CTCs are separated based on the size in a spiral microchannel	[27]
ApoStream^®^	Polarizability	A dielectrophoretic (DEP) force arises when an electric field is applied to the cells with different dielectric characteristics producing the separation of the cells	[28]
The CellSearch^®^ system technology (Menarini-Silicon Biosystems)	Immunomagnetic cell selection system	Antibody-labeled magnetic nanoparticles that target epithelial cell adhesion molecules of CTCs	[29]
AdnaTest		Tumor cell enrichment based on magnetic beads that are coupled to a mixture of antibodies	[30]
MagSweeper		The capture of CTCs using magnetic rods covered with removable plastic sleeves	[31]
CTC-iChip	Microfluidic system	CTC isolation by lateral displacement, inertial focusing, two-stage magnetophoresis, and depletion antibodies against leukocytes	[32]
GEDI chip		The chip is composed of a row of posts coated with antibodies. The larger cancer cells collide with the row of posts and stick on as the other cells flow through	[33]
The OncoCEE^®^ (Biocept, Inc.)		The platform utilizes an antibody capture cocktail and CTCs are captured in a microchannel	[34]
The IsoFlux System™		The CTCs and other rare cells flow through the microfluidic channel and reach an isolation zone where the cells get pulled as they pass through an external magnetic field	[35]
LiquidBiopsy^®^		Cells captured on a flow-chip technology that processes the reagents for the immunomagnetic isolation of target rare cells	[36]
Herringbone-chip or HB-chip		A microvortex mixing device that ensures contacts of cells with antibody-coated surfaces for the capture of CTCs	[37]
GEM chip		The staggered herringbone grooves disrupt streamlines inducing microvortex and capture of CTCs inside the channels	[38]
Graphene oxide (GO) chip		Graphene oxide nanosheets stuck to gold nanoparticles promote the growth of molecular chains that grab onto CTCs	[39]
BioFluidica		Sinusoidally-shaped channels are coated with antibodies, which isolate specific CTCs from whole blood	[40]
NanoVelcro CTC chip		Capture agents and embedded nanosubstrates increases the affinity between CTCs and surface capture	[41,42]
The magnetic ranking cytometry (MagRC)		A microfluidic device sorts the cells into different zones based on magnetic labeling levels	[43]
Gold nanoparticles (AuNPs)		Gold nanoparticles are an efficient platform for assembling multivalent DNA aptamers for high efficiency cell capture	[44]
Microfluidic Western blot		Single-cell resolution Western blot (scWB) to measure a panel of proteins in single CTCs	[45]
Gilupi Nanodetector^®^	Therapeutic apheresis	The nanodetector or thin wire is inserted into the vein of the patient. Cells are docked onto “nano polymer threads” containing antibodies	[46]
In vivo flow cytometry (IVFC)		Quantitative analysis of CTC without the need for the blood collection	[47]

**Table 2:** CTC markers for isolation in different types of cancers.

Cancer Type	Surface Markers	References
Breast cancer	Epithelial cell adhesion molecule, EpCAM	[29,30,56]
	Cytokeratins 7, 8, 18, 19	
	Epidermal growth factor receptor 2, HER2	
	Mucin1, MUC-1	
	Zinc finger protein SNAI1, Snail	
	Neural cadherin, N-cadherin	
	Vimentin	
Castration-resistant prostate cancer	Epithelial cell adhesion molecule, EpCAM	[57,58,59]
	Cytokeratin	
Pancreatic cancer	Epithelial cell adhesion molecule, EpCAM	[31,60]
	Cytokeratins 8, 18, 19	
	Epidermal growth factor receptor, EGFR	
Hepatocellular carcinoma	Epithelial cell adhesion molecule, EpCAM	[32,33]
	Cytokeratin	
	β-catenin	
Melanoma	Melanoma-associated antigen, MLANA	[34]
Small-cell lung cancer	Epithelial cell adhesion molecule, EpCAM	[35,36]
	Chromosome 8 centromere probe, CEP8	
	Synaptophysin, SYP	
	Enolase-2, ENOS	
	Chromogranin A, CHGA	
	Neural cell adhesion molecule, CD56/NCAM	
Non-small cell lung cancer	Epithelial cell adhesion molecule, EpCAM	[37,38]
	Cytokeratins 8, 18, 19	
	Cell surface vimentin, CSV	
Urinary bladder cancer	Epithelial cell adhesion molecule, EpCAM	[39,40]
	Cytokeratins 8, 18, 19	
Ovarian cancer	Epithelial cell adhesion molecule, EpCAM	[41,42]
	Cytokeratin	
Colon and rectum cancer	Epithelial cell adhesion molecule, EpCAM	[18,19,61]
	Cytokeratin	
	Cell surface vimentin, CSV	
Esophageal squamous cell carcinoma	Twist2Cla	[43]
	Pan-cytokeratin (AE1/AE3)	
	Epithelial cell adhesion molecule, EpCAM	
Brain cancer and glioblastoma	Glial fibrillary acidic protein, GFAP	[45,62]
	SRY-related HMG box, Sox2	
	Tubulin beta-3, TUBB3	
	Epidermal growth factor receptor, EGFR	
	A2B5	
	Hepatocyte growth factor receptor, MET	
	OLIG2	

## Data Availability

Not applicable.
